# Constrained Density Functional Theory: A Potential-Based Self-Consistency Approach

**DOI:** 10.1021/acs.jctc.2c00673

**Published:** 2022-09-13

**Authors:** Xavier Gonze, Benjamin Seddon, James A. Elliott, Christian Tantardini, Alexander V. Shapeev

**Affiliations:** † European Theoretical Spectroscopy Facility, Institute of Condensed Matter and Nanosciences, Université Catholique de Louvain, Chemin des étoiles 8, bte L07.03.01, Louvain-la-Neuve B-1348, Belgium; § Department of Materials Science and Metallurgy, University of Cambridge, 27 Charles Babbage Road, Cambridge CB3 0FS, U.K.; | Hylleraas Center, Department of Chemistry, UiT the Arctic University of Norway, P.O. Box 6050 Langnes, Tromsø N-9037, Norway; ⊥ Institute of Solid State Chemistry and Mechanochemistry SB RAS, Novosibirsk 630128, Russian Federation; # Skolkovo Innovation Center, Skolkovo Institute of Science and Technology, Bolshoy Bulvar, 30s1, Moscow 121205, Russia

## Abstract

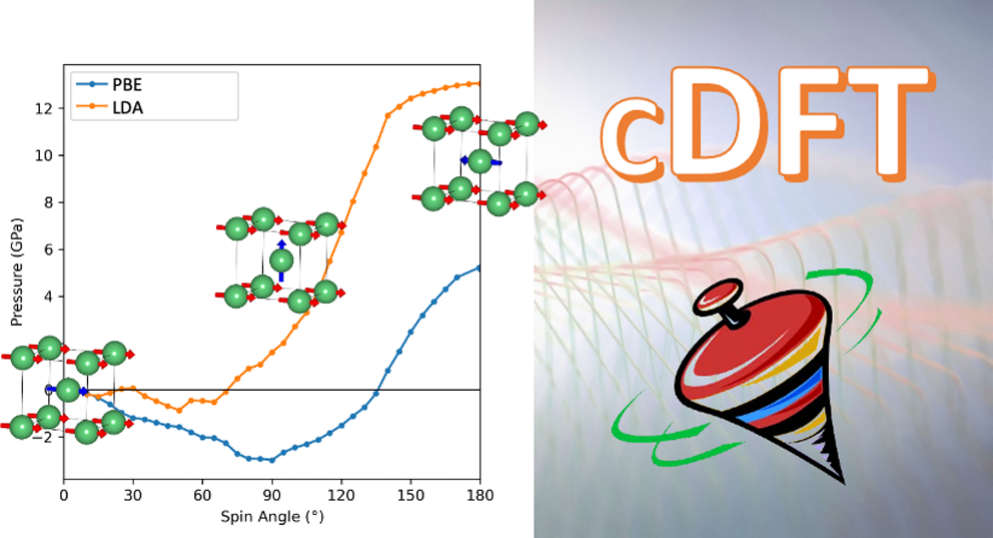

Chemical reactions, charge transfer reactions, and magnetic materials are notoriously difficult to describe within Kohn–Sham density functional theory, which is strictly a ground-state technique. However, over the last few decades, an approximate method known as constrained density functional theory (cDFT) has been developed to model low-lying excitations linked to charge transfer or spin fluctuations. Nevertheless, despite becoming very popular due to its versatility, low computational cost, and availability in numerous software applications, none of the previous cDFT implementations is strictly similar to the corresponding ground-state self-consistent density functional theory: the target value of constraints (e.g., local magnetization) is not treated equivalently with atomic positions or lattice parameters. In the present work, by considering a potential-based formulation of the self-consistency problem, the cDFT is recast in the same framework as Kohn–Sham DFT: a new functional of the potential that includes the constraints is proposed, where the constraints, the atomic positions, or the lattice parameters are treated all alike, while all other ingredients of the usual potential-based DFT algorithms are unchanged, thanks to the formulation of the adequate residual. Tests of this approach for the case of spin constraints (collinear and noncollinear) and charge constraints are performed. Expressions for the derivatives with respect to constraints (e.g., the spin torque) for the atomic forces and the stress tensor in cDFT are provided. The latter allows one to study striction effects as a function of the angle between spins. We apply this formalism to body-centered cubic iron and first reproduce the well-known magnetization amplitude as a function of the angle between local magnetizations. We also study stress as a function of such an angle. Then, the local collinear magnetization and the local atomic charge are varied together. Since the atomic spin magnetizations, local atomic charges, atomic positions, and lattice parameters are treated on an equal footing, this formalism is an ideal starting point for the generation of model Hamiltonians and machine-learning potentials, computation of second or third derivatives of the energy as delivered from density-functional perturbation theory, or for second-principles approaches.

## 1 Introduction

The vast majority of first-principles simulations of ground-state properties of molecules, condensed matter, and nanosystems relies on density functional theory (DFT). However, one is also interested in excited state properties, while, strictly speaking, DFT is a theory for the electronic ground state: the fundamental theorems of DFT rely on a minimization of the energy in the functional space of many-body electronic wavefunctions. The electronic coupling with the external potential being only determined by its electronic density, one demonstrates that the exchange–correlation energy is a unique functional of the ground-state density.^1,2^ For selected classes of low-lying energy states, the same line of thought, based on a minimization principle, has also a strong theoretical basis. For example, taking into account spin magnetization yields spin density functional theory (SDFT). In this case, the exchange–correlation energy becomes a functional of the ground-state density and magnetization.^3^

The space of allowed charge densities or magnetizations might be further constrained, giving access to other low-lying energy states. For example, the charge in some region of space, be it around an atom or on some fragment, might be forced to some predefined value to describe chemical reactions with charge transfer. Similarly, the magnetization vector, or just its direction in the neighborhood of an atom, might be constrained to solve key problems in solid-state chemistry, such as the search for ferromagnetic semiconductors and stable half-metallic ferromagnets with Curie temperatures higher than room temperature. The angular-momentum-projected occupation might also be considered. Such generalizations^4^ should be accompanied by the proper redefinition of the exchange–correlation functional, which should depend explicitly on the constraint. In this case, the formalism, known as constrained density functional theory (cDFT), is as theoretically justified as DFT or SDFT. In practice, though, unlike for DFT or SDFT, the usual functionals are not modified, giving powerful but approximate methodologies to explore the low-lying excited states of systems made of electrons and nuclei.

cDFT has been applied in two major fields of research. Constraining the charge on some molecular fragments allows one to explore the gradual transfer of an electron from one fragment to another and provides parameters for Marcus theory.^5^ Constraining the spin magnetization in the neighborhood of an atom inside a solid allows one to obtain the energy of the system as a function of the local magnetization.^4^ This can be combined with more usual variables governing the energy in first-principle calculations, such as the atomic positions or cell parameters. Thus, cDFT can provide parameters for models of the magnetic state of matter, including the Heisenberg model, with the associated description of magnons,^4,6,7^ or for second-principles models^8−11^ or for constituting training sets for the fitting of machine-learning interatomic potentials.^12−16^

The implementations and applications of cDFT over the years have been numerous and have been reviewed by Kaduk and Van Voorhis in 2012.^5^ In 2016, a list of existing implementations was collected by Melander and co-workers.^17^ Then, one further implementation was described by Hegde and co-workers.^18^

Several methods have been proposed to impose the constraints. In the first one,^19^ an inner “micro”-self-consistency loop is added to the usual DFT self-consistency loop. In this inner loop, the potential (or local magnetic field) is varied to impose the constraint. In the second one,^19^ a penalty function is added to the energy functional. Also, in the specific case of the imposition of the direction of the local magnetization, one can build in directly the constraint in a linearized augmented plane wave formalism,^20^ but this is a specific case. None of these techniques consider the atomic magnetization or the fragment charge on the same footing as the atomic positions or the cell parameters, namely as “external” parameters to the self-consistency problem, for which the same treatment is applied, and with respect to which, the energy and its derivatives are exactly obtained without any restriction.

In the present work, we show that a potential-based self-consistency approach is precisely capable of placing the local magnetization, fragment charge, atomic positions, and strains on a par. We explain the approach on a simple case in which the charge of one fragment is constrained and explain why a similar approach cannot be obtained using a density-based self-consistency approach while usually both are equivalent. Then, we generalize the approach to a combination of constraints, be they fragment charge constraints and/or local magnetization constraints and/or local magnetization directions and/or local magnetization amplitudes. We derive the expressions for the gradients with respect to the value of the constraint (e.g., chemical hardness or spin torque) with respect to the atomic positions (i.e., the forces) and with respect to the strains (i.e., the stress tensor).

The implementation of this approach has been carried out, and we apply it to the iron body-centered cubic (bcc) phase, with two atoms per conventional unit cell. The technique allows one to vary independently the two magnetization vectors by either fixing their value, relative angle, or amplitude and monitor different quantities as a function of such parameters. We first reproduce the magnetization amplitude as a function of the magnetization angle available in the literature in both LDA and GGA and obtain excellent agreement with previously published results, despite different parameters (e.g., the basis of functions or a different projector-augmented wave (PAW) atomic data set). Then, we carry on with the computation of the stress at fixed volume, as well as optimized volume, as a functional of the magnetization angle as well. We also compute cross derivatives of the energy of the system with respect to both difference of charge on the two atoms and magnetization of the two atoms by two techniques: second-order finite differences of total energies and first-order finite differences of analytic hardness and spin torque with excellent agreement.

The theory is presented in Sec. 2, which covers (i) some background information about density- and potential-based DFT self-consistency approaches, (ii) the concepts of potential-based cDFT in the simple case of one constraint, first in a Lagrange multiplier approach and then in a new cDFT functional, (iii) the specification of the types of cDFT constraints, (iv) the treatment of multiconstraint cDFT, and (v) the computation of stress in cDFT. Section 3 presents first the computational details, then proceeds with validation tests against published results, and concludes with the investigation of the stress–magnetization relationship and the charge transfer–magnetization relationship for bcc iron in the cDFT formalism.

## 2 Theory

In this section, we highlight first the conceptual basis of density- or potential-based DFT self-consistency at the heart of the vast majority of DFT calculations worldwide. We then show how the potential-based self-consistent method can be generalized to cDFT for the simple case of one constraint applied to the density (imposing the charge of a fragment). The corresponding chemical hardness is obtained, as well as the expression of first-order derivatives with respect to modification of the external parameters (Hellman–Feynman theorem). Then these equations are generalized to multiple constraints, possibly defined in overlapping regions, and applied to both charge and magnetization. The generalized expressions for the chemical hardness, spin-torque, forces, and stresses are then presented.

### 2.1 Density- and Potential-Based DFT Self-Consistency Approaches

Consider a set of electrons placed in a potential external to the electron system, *v*_ext_, sum of the nuclei potentials (or ionic pseudopotentials), and other external potential applied to the electron system. The DFT energy  is expressed as a function of occupied orthonormal Kohn–Sham wavefunctions, {*ϕ*_*i*_}, where *i* labels occupied states with occupation number *f*_*i*_ (e.g., *f*_*i*_ = 2 for doubly occupied orbitals, spin up and spin down) and includes the kinetic energy, potential energy of the electrons, and the density-dependent Hartree and exchange–correlation energy *E*_Hxc_[*ρ*]
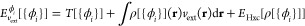
1with kinetic energy and electronic density given by
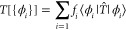
2and
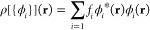
3where *T̂* is the kinetic energy operator.

Self-consistency can be formulated as requiring the wavefunctions to minimize 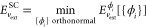
4Indeed, constrained minimization of eq 4 through the Lagrange approach yields the well-known Kohn–Sham equations and associated self-consistent requirement of density, potential, Kohn–Sham Hamiltonian, and wavefunctions. Explicitly, for any given charge density *ρ*, the screened potential is obtained as
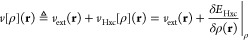
5In a similar way, for any given trial-screened potential denoted *u* and the associated local potential operator *û*, the corresponding Schrodinger equation is solved
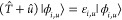
6and the resulting wavefunctions, |ϕ_*i*,*u*_⟩, inserted in the density expression eq 3, delivers the density as a functional of the potential noted *ρ*^v^
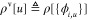
7

The self-consistent density ρ* thus fulfills
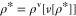
8In the latter equations, the density, potential, and wavefunctions are functions of the position. For the sake of clarity, their position dependence, as in eq 3, has not been explicitly mentioned, as in most of the following equations.

Many iterative techniques have been developed over the years to tackle the self-consistency problem.^21−24^ A trial input density at step *n*, *ρ*_*n*_^in^, delivers an output density *ρ*_*n*_^out^
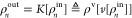
9

The discrepancy between the output and input densities
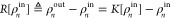
10is usually referred to as the density residual. The vast majority of algorithms to solve this self-consistency problem relies on the knowledge of pairs of trial density and the corresponding residual to infer the next trial density. The easiest algorithm to implement, that is, simple mixing, is defined by
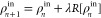
11with *λ* being a tunable parameter. Most sophisticated algorithms take advantage of the history (at least the most recent part of it) and possibly include some preconditioning operator *P*, even varying at each step
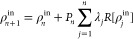
12where the set of parameters and the possible preconditioner are computed on the flight from the history and differ for different algorithms.

Instead of such density-based mixing approaches, potential-based mixing approaches can also be found in the literature.^22^ In order to distinguish the (nonlinear) operators appearing in this approach from those appearing in the density-based approach, we label them with a “v” superscript. In the potential-based approaches, instead of eqs 8–12, one relies on
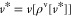
13
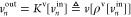
14
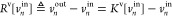
15
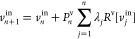
16

The density- and potential-mixing approaches are dual to each other: in the case of usual (unconstrained) DFT, for each density-based mixing algorithm, there exists an equivalent potential-based mixing algorithm in which the pairs of density and the corresponding density residual are replaced by pairs of potential and the corresponding potential residual.

This duality does not extend to all characteristics of these two approaches. Indeed, one can immediately associate to a given screened potential *u*, taken as trial potential, a set of wavefunctions {*ϕ*_*i*,*u*_}, through eq 6. On the contrary, there is no such set of wavefunctions immediately associated with every trial density, *ρ*, even if one generates such wavefunctions through the screened potential *v*[*ρ*]—unless one is at self-consistency.

Focusing on the potential-based approach, the self-consistent electronic energy expression, eq 4, is straightforwardly recast as a minimization problem in the space of trial screened potentials as follows
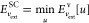
17with
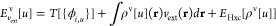
18

The gradient of this functional of the potential has been computed in ref (25)
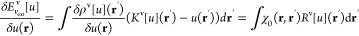
19where the independent-particle susceptibility *χ*_0_(**r**, **r**′) is to be evaluated at the screened potential *u*. This gradient obviously vanishes at the minimum since *R*^v^[v*] vanishes. In practice, multiplication by *χ*_0_^–1^, like in ref (25), delivers a preconditioned gradient, which is nothing else than the residual *R*^v^[*u*], so that *χ*_0_(**r**, **r**′) does not even have to be computed. Hence, this approach shows that the usual potential-based self-consistency algorithms, eq 16, can be understood as mixing of the preconditioned potential gradients of the electronic energy eq 18 from the current and previous steps. Note that an even better preconditioner can be defined if the inverse dielectric constant is known ( also see ref (25)).

As a side note, the present formulation of cDFT shares with the OEP method^26−29^ the usage of the screened potential as the fundamental object to be varied in order to optimize a variational expression. In the OEP case, there is no such constraint as in cDFT, although the OEP variational expression is formulated not only in terms of density (and magnetization) but also in terms of orbitals.

### 2.2 Imposing the Charge of One Fragment in cDFT through the Lagrange Approach

Let us present the concepts of the potential-based self-consistent approach to cDFT for the simple case of one constraint, namely constraining the weighted charge of one fragment, labeled generically as “A.” The weighted charge on fragment A, a functional of *ρ*, is defined as follows
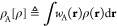
20for some weight function *w*_A_(**r**), spanning the region A where the fragment is located, typically *w*_A_(**r**) = 1 well inside this region and *w*_A_(**r**) = 0 outside, so *w*_A_ smoothly decreases to zero when reaching the frontier of A. Mathematically, the constraint of fragment charge being *N*_A_ is formulated as
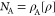
21

Such a constraint might be dealt with by adding a penalty function multiplied by a weight, as in refs (30) and (31). In the limit of infinite weight, the constraint is exactly fulfilled. Unlike asserted in ref (31), this formulation is not a Lagrange multiplier approach. Anyhow, this technique is plagued with numerical instabilities and definitely does not treat the values of the constraint similarly to other external parameters, such as atomic positions or cell parameters.

By contrast, in the Lagrange multiplier method, the energy is augmented by the product of a Lagrange multiplier Λ with an expression that vanishes when the constraint is fulfilled. The proper choice of the Lagrange multiplier makes the constraint exactly satisfied. The cDFT electronic energy, dependent on the Lagrange multiplier, is the augmented functional
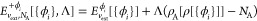
22for which self-consistency can be formulated similarly to the DFT case, eq 4, as
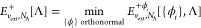
23

The minimization procedure delivers wavefunctions and density as a function of Λ (also *v*_ext_ and *N*_A_), and the final choice of Λ is the one that yields fulfillment of the constraint. Enforcing the value of Λ that satisfies the constraint can be done along the iterative self-consistent procedure by using microiterations, as proposed by Wu and Van Voorhis.^19^ However, again, this does not treat the variable *N*_A_ on the same footing as other external variables, such as the atomic positions or cell parameters. Moreover, the algorithms to be used differ from the ones for a usual self-consistency loop without microiterations, and there is an overhead associated with such treatment.

The potential-based approach can be adapted as well in order to include similarly a Lagrange augmentation. This will prove more fruitful. The augmentation is as follows
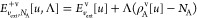
24where *ρ*_A_^v^[*u*] is a shorthand for *ρ*_A_[ρ^v^[*u*]], and where self-consistency is reached at the minimum over all trial potentials
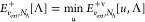
25

In eq 24, the gradient of  with respect to the screened potential *u* is given by eq 19, and a similar approach delivers the gradient of the entire  with respect to *u*
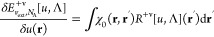
26with
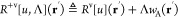
27

According to eqs 25 and 26, a self-consistent solution is obtained for *u* = v* that satisfies
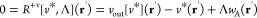
28for all **r**′. Namely, it occurs when the difference between the output and input potentials is a multiple of the weight function, the prefactor being the Lagrange parameter.

In particular, multiplying this equation by *w*_A_(**r**′) and integrating over **r**′ allows one to obtain the value of Λ that makes the residual vanish
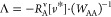
29where
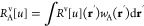
30and
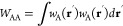
31

This constitutes a proper mathematical formulation of potential-based cDFT within the Lagrange multiplier approach. Moreover, in this potential-based approach, the Lagrange parameter is immediately determined, unlike in the Wu and Van Voorhis approach.^19^ This is due to the simple relationship between the potential-based residual and the weight function, eq 28, for which there is no simple equivalent in the wavefunction- or density-based cDFT formulations.

### 2.3 Simple Potential-Based cDFT Functional

In order to go one step further, a new cDFT functional, *E*^cDFT^, which admits the same self-consistent solution as eq 23 or 25, is introduced. The Lagrange parameter in eq 24 is replaced by the expression eq 29 evaluated at *u* instead of v*, giving
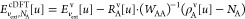
32

This new functional places *v*_ext_ (in which the atomic positions and cell parameters enter) and *N*_A_ on the same footing, namely as external parameters of the calculation. Still  is a functional of the screened potential *u* only, without auxiliary Λ to be determined. By construction, at the self-consistent v* for the given *v*_ext_ and *N*_A_, the functional has the same value as the cDFT functional based on the Lagrange parameter, delivering the self-consistent value of the electronic energy
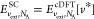
33In this equation, one has not explicitly mentioned the v* dependence on *v*_ext_ and *N*_A_. Equation 33 is stationary with respect to variations of *u* around v*
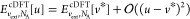
34

The gradient of this functional with respect to *u* is
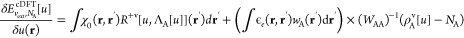
35where
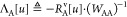
36and *ϵ*_*e*_(**r**, **r**′) is the electron dielectric response function. The gradient vanishes when *u* = v* since in this case, both *R*^+v^[v*, Λ_A_[v*]] and  vanish. This actually proves the stationary character of  at *u* = v*.

Importantly, Λ_A_[*u*] is the precise value that makes *R*^+v^[*u*, Λ_A_[*u*]](**r**′) orthogonal to *w*_A_
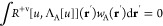
37

To demonstrate this assertion, insert eq 36 in eq 27 and integrate. This suggests treating the two parts of the gradient with different preconditioning, eq 35. The following expression, obtained by removing *χ*_0_ from the first term and *ϵ*_*e*_ from the second, can indeed be used to define a residual for the cDFT
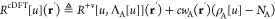
38Since the first and second terms belong to orthogonal subspaces, the residual *R*^cDFT^ vanishes for all **r**′, only if both *R*^+v^ and  vanish, which amounts to obtain self-consistency, as, on the one hand, eq 28 is fulfilled, and, on the other hand, the constraint eq 21 is imposed. In expression eq 38, *c* is a constant whose value is formally arbitrary but for practical purposes should be of order one, as it defines the balance between the convergence inside the space spanned by *w*_A_ and the convergence inside the space perpendicular to it. This formulation of a residual for cDFT opens the door to the adaptation of all algorithms used for potential-based DFT self-consistency.

Since the new functional  is stationary, its behavior with respect to modifications of parameters *v*_*ext*_ and *N*_A_ fulfills the 2*n* + 1 theorem of perturbation theory,^32^ allowing to obtain easily numerous derivatives of the total energy^33−39^ with respect to changes in the parameters of the calculation: at first order, forces, and stresses but also chemical potential and spin-torque (see later), specifically for cDFT; at second-order interatomic force constants (yielding vibrational frequencies), Born effective charges, and elastic constants but also cross-derivatives between atomic displacements, local magnetization, and fragment charges, specifically for cDFT.

In particular, in first order, the derivative with respect to the fragment charge *N*_A_, that is, the chemical potential of fragment A,^40^*μ*_A_, is
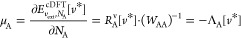
39This derivation highlights relations between different quantities appearing in the formalism. For the sake of simplicity, we will often use *μ*_A_ to denote these different quantities.

One also recovers Hellmann–Feynman theorem,^41,42^ a specific instance of the 2*n* + 1 theorem. This gives, for example, the force exerted on atom *κ* in direction *α* as
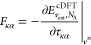
40where *τ_κα_* is the coordinate atom *κ*. When taking the derivative, the implicit dependence of v* on *τ_κα_* is not to be taken into account, according to the Hellmann–Feynman theorem.

The dependencies of  on τ_κα_ occurs through the external potential *v*_ext_ and the weight function *w*_A_. Since the second term in eq 32 does not depend explicitly on *v*_ext_ and the first term does not depend on *w*_A_, one gets
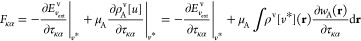
41the first term is the usual DFT expression for the force, albeit evaluated at v*, that is determined under the constraint eq 21. The second contribution is easily evaluated once the density has been self-consistently determined. Forces are thus byproducts of the self-consistent calculation, as usual in normal DFT. Note that while *R*_A_^v^ and *W*_AA_ in eq 32 depend on the atomic position, their contribution to the force vanishes, as the second line in eq 32 contains ρ_A_^v^[*u*]–*N*_A_, which vanishes at *u* = v*. Other derivatives with respect to parameters for the DFT calculation can be obtained likewise.

### 2.4 Types of cDFT Constraints

The previous approach, presented for the case of the specific constraint of imposing the charge of a fragment, can be generalized to several simultaneous constraints and constraints more general than fragment charges. Such possible constraints have been discussed in ref (5) and other references presented in the introduction. While the original DFT approach considered a functional of the charge density only, later generalizations introduced functionals of collinear magnetization or even noncollinear magnetization, both equivalently formulated in terms of the spin-density matrix. The spin-density matrix *ρ*_*ss′*_[{*ϕ*_*i*_}](**r**) can be computed from spinorial wavefunctions {ϕ_*si*_(**r**)}, with *s* and *s*′ subscripts being up (↑) or down (↓)
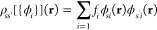
42Constraints might be defined in terms of linear combinations and integrals of the spin-density matrix elements, for example,
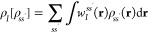
43The  function has to be specified for each possible value of the index *I*, possibly a composite index, characterizing the different constraints.

For example, and in view of practical applications later, the magnetization along *x* around atom *κ*, *M*_*xκ*_, might be imposed by requiring the following constraint
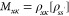
44with the weight function inside eq 43 being
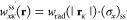
45in this expression, **r**_*κ*_ ≜**r** – ***τ***_*κ*_, *σ*_*x*_ is the 2 × 2 Pauli matrix for the *x* direction, and *w*_rad_(*r*) is a radial weight function (e.g., *w*_rad_(*r*) is 1 for *r* smaller than some cut-off radius *r*_*c*1_, then decreases smoothly beyond that radius, and becomes exactly zero beyond some other cut-off radius *r*_*c*2_). An alternative formulation, more convenient for numerical evaluation and computation of forces and stresses, uses
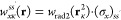
46with the following obvious relation *w*_rad_(*t*^1/2^) = *w*_rad2_(*t*).

The constrained magnetization along *y* or *z* for the same atom, as well as for other atoms, can be defined similarly to eqs 44 and 45. All these constraints can be considered together.

We will also consider constraining only the direction of magnetization, using a linear formulation as well, like in ref (20). Let ***ê*** be a unit vector along the constraint direction for the magnetization, the directional constraint can be obtained by requiring together
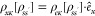
47
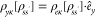
48
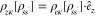
49with
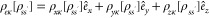
50The function *ρ*_*eκ*_[*ρ*_*ss*′_], as well as its *x*, *y*, *z* counterparts, is linear in *ρ*_*ss*′_ and thus also the constraints (eqs 47–49). This constraint will be illustrated in the application part.

Finally, even nonlinear constraints might be considered. For example, the amplitude of the magnetization vector for atom *κ*, ∥*M*_*κ*_∥, can be imposed by requiring
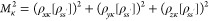
51This has also been implemented and tested but will not be illustrated. The Lagrange multiplier method also deals easily with such nonlinear constraint, as well as the potential-based cDFT formulation.

### 2.5 Multiple Constraints in Potential-Based cDFT

Now, we generalize most of the equations in Sections 2.2 and 2.3 to the case of several constraints and constraint types. The indices *I* or *J* run through the whole set of constraints and replace the index A that we had used in these sections to explain the concepts in the case of one fragment.

For the target value of constraint *I*, we use the notation *N*_*I*_ generically, even if it is a magnetization-type constraint. Like the density that becomes the spin-density matrix, the potential (screened or external) and the residual both become two-by-two spin matrices. The notation might become very cumbersome so that we do not explicitly mention the two spin variables when not strictly needed, and also we combine the two-spin labeling *ss*′ into one label *S* placed as superscript. Therefore, we use *v*_ext_^*S*^ or even *v*_ext_ instead of  and, likewise, *u*^*S*^ or *u* instead of *u*^*ss*^′^^ and *R*^*S*^ or *R* instead of *R*^*ss*^′^^. By contrast, for this multiple-constraint generalization, we explicitly treat the indices *I* or *J*.

For each constraint, there is a Lagrange multiplier Λ_*I*_. The augmented energy eq 24 becomes
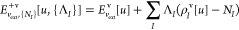
52with
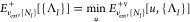
53

The generalization of the self-consistent solution defined by eqs 27 and 28 is as follows. For the self-consistent v*, the condition is
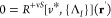
54for all **r**′ with definitions
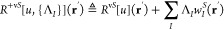
55and
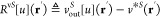
56Equation 54 must be true for all values of *S* and **r**′.

Multiplying these equations by  for all values of *J*, then integrating over **r**′ and summing over *S* allows one to obtain the value of Λ_*I*_ that makes the residual vanish
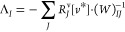
57where
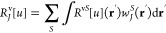
58and
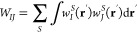
59The appearance of the cross-constraint matrix *W*_*IJ*_ and its inverse is key to the formulation of a many-constraint potential-based cDFT functional
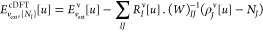
60For the self-consistent v*, one recovers
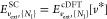
61and  is stationary with respect to variations of *u* around v*
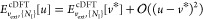
62as in eq 34. This is the central result of this work.

Thus,  possesses many of the properties enjoyed by usual DFT functionals, in particular, the possibility to apply the 2*n* + 1 theorem of perturbation theory, including the Hellmann–Feynman theorem. It is also clear that the constraints (fragment charge, fragment magnetization, and variation thereof) are treated on the same footing as other parameters of the problem that enter the play through the external potential, such as atomic positions, cell parameters, or applied external fields.

The following residual can be used to perform searches for self-consistency, with usual algorithms
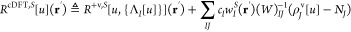
63Indeed, the first term lives in a subspace orthogonal to the second term.

The derivative of  with respect to the value of the constraint *N*_*J*_, evaluated at the self-consistent screened potential, v*, is given by
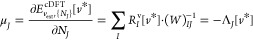
64The same notation *μ*_*J*_ as for the derivative of the fragment charge is used although such derivative might correspond to a rather different physical phenomenon. For example, when the constraint imposes a magnetization direction, such a derivative is the spin torque, namely the gradient of the energy with respect to a change of the direction of the spin magnetization, for example, the torque that is needed to ensure that the magnetization is strictly constrained to a given value.

The force eq 40 becomes
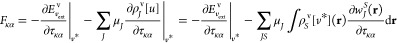
65If the weight functions decompose, as in eq 46, namely if they are a product of a rigid spherical function attached to atom *κ*_*J*_ position, times a spin-dependent quantity *Q*_*J*_^*S*^, independent of **r** and *κ*
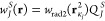
66then
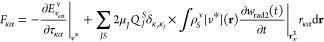
67Note the presence of the  factor: with the weight functions, as in eq 66, only the rigid spherical function attached to atom κ will contribute to the force correction. This is not true in general since modification of the atom *κ* position might induce modification of the weight function linked to another atom.

Such a weight function (eq 66) is commonly used for computing local magnetization. In the case of a real space evaluation of the integral in eq 67, on a grid of points, the decrease in the cut-off function 1 to 0 should not be too steep; otherwise, the numerical evaluation of the space integral of the derivative in such an equation will have large numerical noise (and error). The derivative of the function *w*_rad2_(*t*) is accompanied by a *r*_*κα*_ factor.

An equivalent formula is obtained after considering that the derivative with respect to *τ_κα_* is equal to the negative derivative with respect to the position *r*_*α*_, then integrating by parts
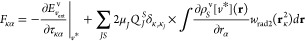
68

Evaluation of the density derivative in the Fourier space then transform to real space might yield smaller numerical noise than the previous procedure based on eq 67 but has not been implemented.

Another type of weight function makes sense in the cDFT context: partitioning in regions around atoms, which completely paves the entire space so that the charge density is allocated to one or another atom. For example, Bader,^43^ Hirshfeld,^44^ or Becke^45^ partitioning yield
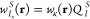
69where for each **r** in the full space
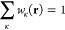
70the sum running over all atoms in space. Also, in this case, for an evaluation in real space, the weight function cannot decrease abruptly from 1 to 0 in order to compute the forces. Thus, the *w*_*κ*_(**r**) functions overlap. The present formalism easily deals with such cases by means of nonzero off-diagonal overlap elements *W*_*IJ*_ (see eq 59). The implementation of the Becke partitioning and associated forces has been described in detail in ref (46).

### 2.6 Stress in cDFT

Although the implementation of forces is common in cDFT, the implementation of stress has not been reported to our knowledge. The stress tensor, *σ_αβ_*, where *α* and *β* are along the three Cartesian directions, is obtained as the derivative of the energy per unit cell of volume, which we will note as *E*_Ωtot_^SC^ with respect to the deformation tensor *η_αβ_* divided by the cell volume Ω.^47,48^ In our notations
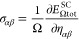
71where the deformation tensor is such that the position vector *r*_α_ becomes *r_α_* = *r_α_* + *∑_β_η_αβ_*r_*β*_. Similarly,  will be the energy per unit cell obtained from *v*_ext_ in the potential-based self-consistent approach.

With respect to the previous formalism, treating the periodic case will explicitly assume that each constraint *J* is repeated periodically in every primitive cell. In order to have the cell contribution of constraints to the total energy per primitive cell, the summation over constraint *J* will be restricted to one instance of each periodically repeated constraint.

The cDFT stress is then written as
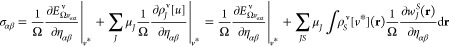
72In the case of atom-centered, separable weight functions, such as eq 66, the stress becomes
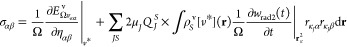
73The derivative of the function *w*_rad2_(*t*) is accompanied by a *r*_*κα*_ factor and *r*_*κβ*_ factor, while the contribution of all constraints is summed.

The applications in the next section rely on this formula. An alternative formulation of the stress, similar to the one for the forces, eq 68, is possible but has not been tested.

## 3 Results and Discussion

In support of the concepts presented in the theory section, we provide validation tests against known results, as well as demonstrate the usage of the potential-based cDFT functional to investigate stress-magnetization and charge-magnetization couplings, for the paradigmatic case of BCC iron.

### 3.1 Computational Details

The potential-based cDFT approach has been implemented in ABINIT.^49,50^ Results presented in this section have been obtained with publicly available version 9.6, except for some fixes needed to compute the stress, which will be made publicly available in ABINIT v9.8. The cDFT electronic energy (eq 60) is optimized using Pulay residual minimization algorithm,^21^ keeping seven past pairs of trial potential and corresponding residuals (eq 63) in the history. Other algorithms are also available in ABINIT but are not demonstrated hereafter. We have observed several cases in which the Pulay residual minimization algorithm does not yield convergence with the present cDFT formalism and its implementation. This only occurred for noncollinear magnetization calculations within GGA(PBE) and not for LDA. We do not report these cases in the present work, as they will be the subject of further work.

The representation of wavefunctions relies on the PAW formalism.^48^ Two PAW atomic data are tested, the first one using the LDA exchange–correlation functional for comparison to the work by Kurz et al.^20^ and another one using the GGA-PBE^51^ exchange–correlation functional for all other calculations. The pseudopotential cut-off radius *r*_c2_ = 1.065 Å is used as the cut-off radius for the definition of the atomic spins and charges. The width of the smearing region is 0.026 Å or roughly 2.5% of the atomic radius. The smearing width is kept small so that comparisons could be made to the Ma and Kurz papers, where muffin tin potentials are used. Still, the smearing width needs to be large enough in order to avoid the numerical instabilities in the pressure calculations, as mentioned in the Theory section. The smeared function, going from 1 to 0, is the inverse of Eq. (B4) of ref (38).

All calculations are performed for a two-atom BCC iron conventional unit cell. For a given *θ* angle between magnetization directions on the two atoms, magnetization on atom 1 is imposed as *M* (sin(*θ*/2), 0, cos(*θ*/2)), while magnetization on atom 2 is imposed as *M* (−sin(*θ*/2), 0, cos(*θ*/2)). The parameter *M* is freely optimized by ABINIT. Among the existing symmetry operations, a binary symmetry axis, exchanging atoms 1 and 2, is present for such calculations and is actually critical to reaching some of the results presented below. Indeed, without such symmetry operation, constraining the magnetization angle *θ* for different atoms using homogeneous constraints Equations 47–50 works if such an angle is lower than 90° but is inherently problematic when a *θ* angle beyond 90° is aimed at. For example, imposing magnetization on atom 1 to be *M*_1_(001) and *M*_2_(sin(*θ*), 0, cos(*θ*)) induces spontaneous switching of *θ* larger than 90° to a value 180° – *θ*, smaller than 90°.

The self-consistency algorithm easily achieves more than six-digit accuracy on the constraint, be it a magnetization component, a magnetization direction or amplitude, or a local charge, so essentially perfectly imposing the constraint.

For comparison with results from previous publications, we use the same lattice parameters: 2.789 Å for comparison with Kurz et al.^20^ and 2.83 Å for comparison with Ma et al.,^31^ respectively. For all other PBE calculations, we use the lattice parameter 2.845 Å obtained from ABINIT relaxation.

A cut-off energy of 30 Ha is used, with a 16 × 16 × 16 grid to sample the Brillouin zone and an electronic smearing of 0.0005 Ha. This is sufficient to converge the energy, spin magnitude, pressure, and transverse spin force.

It is worth noting that the longitudinal value of the spin force, obtained when the magnitude of the spin vector is also constrained, requires a 72 × 72 × 72 grid to sample the Brillouin zone in order to reach convergence. However, this value can be significantly reduced when a nonzero electronic temperature is used.

### 3.2 Validation of the Self-Consistency Approach

**Figure 1:**
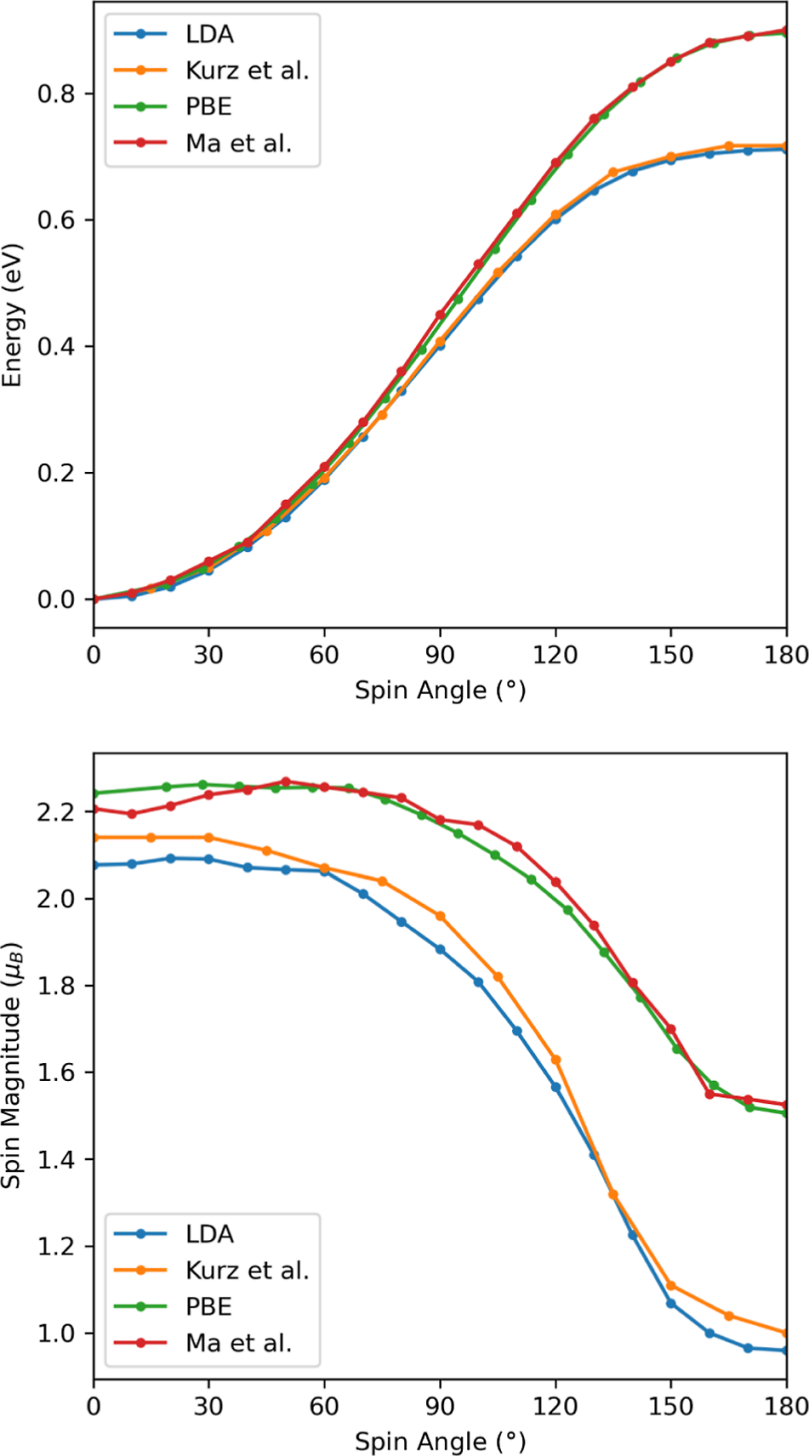
Comparison of energies (top) and the spin magnitude (bottom) as a function of the angle between spin directions. Potential-based cDFT LDA data (blue) are to be compared to those from Kurz et al.^20^ in orange, while potential-based PBE data (green) are to be compared to those of Ma and Dudarev.^31^ in red.

In order to validate the potential-based cDFT method, we compare results with the implementations reported by Ma and Dudarev,^31^ who use the PBE functional, as well as Kurz et al.,^20^ who use the LDA functional. We calculated the variation in energy and spin magnitude as the angle between the spin (or local magnetization) vectors was varied from 0° (ferromagnetic) to 180° (antiferromagnetic) in increments of 10°. The energy and spin magnitudes are plotted in Figure 1 and constitute a convincing validation of the potential-based cDFT implementation. We use a cut-off radius of 1.065 Å for our definition of the atomic spin vector compared to 1 Å for Ma and Dudarev and 1.19 Å for Kurz et al. The slight difference between our value for the radius and that used by Kurz et al., as well as the different PAW atomic data set, explains the rather small albeit non-negligible difference with these calculations. Our values for the spin magnitude are consistently slightly lower than their values. However, this difference is unsurprisingly small since it is the localized d electrons that contribute to the atomic magnetic moment.

The behavior of the spin magnetization as a function of spin angle is not very smooth, albeit continuous, which is in line with the results obtained in previous studies. This is observed despite the fact that the numerical accuracy has been pushed to a high level (e.g., one part per million for the spin magnitude at a given spin angle). In our opinion, this jagged behavior is to be linked to the existence of critical points in the electronic density of states, these being affected by the spin angle hence affecting the spin magnitude.

### 3.3 Stress-Magnetization Coupling

As an example of the strong magnetoelastic coupling in iron, we calculated the pressure for varying spin angles when the cell is fixed, then relaxed the lattice parameters and obtained the variation in the equilibrium lattice parameter. The pressure is minus the trace of the stress tensor *σ_αβ_* (see eq 73).

**Figure 2:**
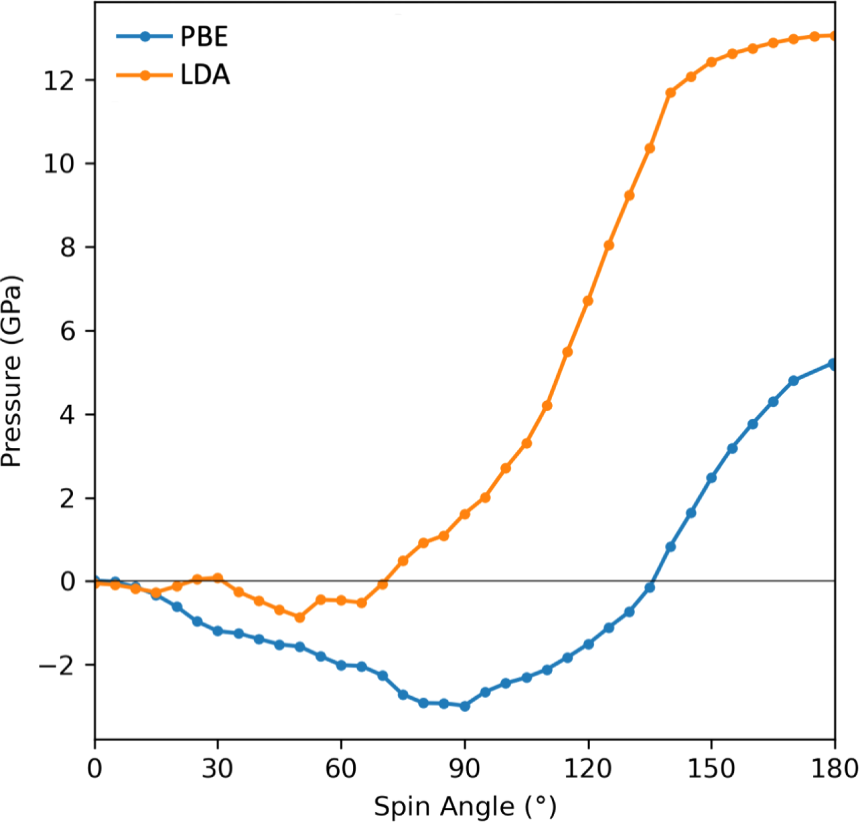
Pressure as a function of the spin angle between the two atoms in a Fe BCC conventional cell using the PBE and LDA exchange–correlation functionals. The lattice parameters are fixed to those found by relaxing the ferromagnetic cell, giving 2.83 and 2.76 Å for the PBE and LDA functionals, respectively.

In Figure 2, the pressure varies within a range of roughly 8 and 12 GPa for the PBE and LDA calculations, respectively, as the spin vectors are rotated between the ferromagnetic and antiferromagnetic configurations. To put this in context, the bulk modulus of iron is 166 GPa.^52^

**Figure 3:**
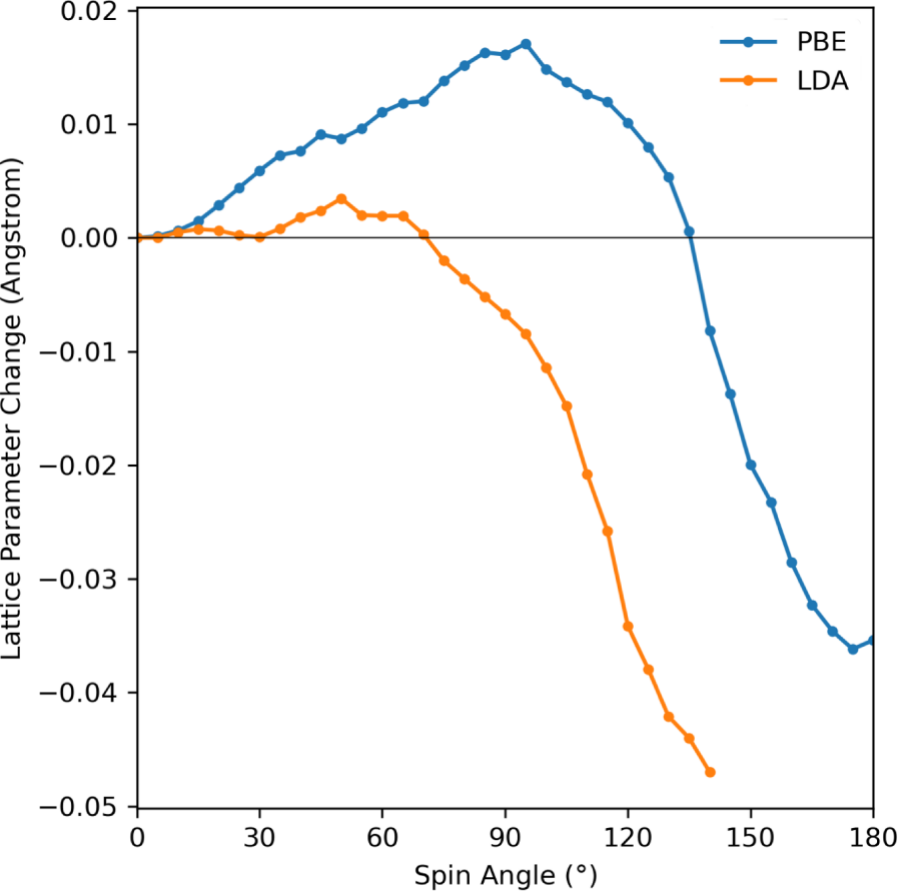
Lattice parameter after structural relaxation of a 2 atom BCC iron unit cell as the angle between the spin vectors is varied. The LDA calculations past 145° started converging to a zero spin configuration and were not included in the plot.

The variation in the relaxed values for the lattice parameters shown in Figure 3 mirrors the pressure changes. The lattice parameter variation is 0.06 Å or roughly 2% of the total lattice parameter, which again demonstrates how changes in the spin configuration can induce significant strains. In order to perform this calculation, the stress obtained at a fixed spin angle was relaxed using cell optimization algorithm in ABINIT. However, it was also independently checked that for a fixed spin angle, the minimum of the total energy as a function of the lattice parameter does indeed correspond to the stress going to zero. A jagged behavior of the pressure and lattice parameter as a function of the spin angle is observed, similar to the spin magnetization of the previous subsection.

### 3.4 Atomic Magnetization and Charge Transfer as Independent Variables

As a demonstration of the combined usage of charge and spin constraints, which will be relevant to addressing joint charge and spin ordering in materials such as rare-earth ferrate systems,^53^ we calculate the Hessian for a 2-atom Fe BCC unit cell, where the variables considered are the two collinear atomic spins and the difference in the charge between the atoms. The derivative of the energy with respect to the charge difference is calculated as
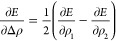
74where Δ*ρ* = *ρ*_1_ – *ρ*_2_ is the charge difference, and the derivatives with respect to the atomic charges are available as the Lagrange multipliers for the charge constraint. The data are presented in Table 1. These second derivatives have been computed by both a second-order centered finite difference method from the total energies, as well as from finite differences of analytical first derivatives. Agreement between these computations is at the level of the number of digits shown in the table.

**Table 1:** Elements of the Hessian for the Energy of a 2-Atom Fe BCC Unit Cell Based on Three Variables^a^

Hessian element	Value
∂^2^*E*/∂*s*_1_^2^	0.02382 Ha μ_B_^–2^
∂^2^*E*/∂*s*_2_^2^	0.02382 Ha μ_B_^–2^
*∂*^2^*E*/*∂*Δ*ρ*^2^	0.21424 Ha *e*^–2^
∂^2^*E*/∂*s*_1_∂*s*_2_	0.00572 Ha μ_B_^–1^ *e*^–1^
∂^2^*E*/∂*s*_1_∂Δρ	–0.01383 Ha μ_B_^–1^ *e*^–1^
∂^2^*E*/∂*s*_2_∂Δρ	0.01382 Ha μ_B_^–1^ *e*^–1^

a The spin magnitudes for atoms 1 and 2 are *s*_1_*s*_2_, respectively, and Δ*ρ* is the charge difference between atom 1 and atom 2.

All the diagonal entries are positive, which is a prerequisite for the stability of the system with respect to spontaneous symmetry breaking. The negative value for ∂^2^*E*/∂*s*_1_∂Δ*ρ* can be understood intuitively as a consequence of spin polarization becoming easier as the amount of electron density, which can be polarized, increases.

To our knowledge, this is the first case in which cDFT has been used with both charge and spin constraints, while the study and discovery of new multiferroic materials^54^ and the analysis of spin and charge orderings^53^ will benefit from such possibility.

Our formulation of cDFT also allows one to develop magnetic machine learning potentials^12−16^ —potentials whose functional form is extended to depend on magnetization (norm, but also direction) and/or atomic charge values in addition to atomic relative positions. More specifically, the “usual” machine-learning potentials define the interatomic interaction energy as a function of the type *T_κ_* and position *τ_κ_* of each atom *κ*. Then, the generalized machine-learning potentials might include the dependence of the energy on the variables presented in Section 2, namely *N_κ_* and/or *M*_*xκ*_, *M*_*yκ*_, *M_zκ_*. Because the proposed cDFT defines a strictly conservative force field as a function of such coarse-grained degrees of freedom, it can be used as the first-principles basis to generate such generalized magnetic machine learning potentials.

In the same spirit, computing the total energy as a function of absolute atomic displacements with respect to a reference unperturbed state, together with the local magnetization and/or charge, allows for the generalization of second-principles models^8−11^ beyond the current ferroelectric materials, to deal with multiferroic materials, for example, as a function of temperature. In both the machine learning potential and the second-principles model cases, the knowledge of the various first-order derivatives for which we have detailed the expressions in Sections 2.5 and 2.6 might prove to be an enabling feature.

## 4 Conclusions

In this work, we have formulated cDFT with a Lagrangian multiplier approach and used the potential as a fundamental variable, allowing us to recast the associated self-consistency problem in a form suited for the application of standard self-consistency algorithms. The potential residual has two components, one directly related to the constraints, which could be on local atomic density or magnetization or both, and the other stemming from the usual definition, which invokes the difference between the input and output potentials albeit projected on a subspace perpendicular to the constraint. This allows one to avoid both (i) the use of a penalty function, which delivers a biased solution to cDFT, and (ii) an additional internal loop, which departs from the usual SCF algorithms.

A simple potential-based cDFT functional, valid for all kinds of constraints placed on the density or spin-density in arbitrary regions of space, has been introduced and shown to be stationary with respect to trial-effective (spin-)potential variations. The powerful 2*n* + 1 theorem of perturbation theory can thus be applied in such a context, allowing the cDFT predictive capabilities similar to its DFT counterpart.

We have also provided the analytic cDFT expression for the derivatives with respect to the constraints (e.g., the local chemical potential or spin torque), as well as for the atomic forces and stress. We have validated the concepts of this approach by their implementation in open-source ABINIT code and then by comparison with published results for the paradigmatic case of Fe BCC. The investigation of stress-magnetization coupling and charge-magnetization coupling has been done as well. In such a context, the atomic spin magnetizations, local atomic charges, atomic positions, and lattice parameters are on an equal footing, which is an ideal starting point for the generation of model Hamiltonians for second-principles approaches and generating training data sets for machine-learning interatomic potentials.

The domain of application of our approach is thus large, even more given the development of new fields of research in which the different perturbations of the bulk or nanostructures are combined, be them electric, magnetic, and stress or its gradient, as testified by the interest in multiferroic materials, flexoelectricity or flexomagnetism, or in materials where charge, spin, and lattice degrees of freedom are coupled to each other. Furthermore, it is applicable to the development of machine learning potentials for crystal structure prediction of magnetic materials.
